# Presentation of Various Tactile Sensations Using Micro-Needle Electrotactile Display

**DOI:** 10.1371/journal.pone.0148410

**Published:** 2016-02-04

**Authors:** Mayuko Tezuka, Norihide Kitamura, Kohei Tanaka, Norihisa Miki

**Affiliations:** 1 Department of Mechanical Engineering, Keio University, Yokohama, Kanagawa, Japan; 2 JST PRESTO, Chiyoda, Tokyo, Japan; University of Chicago, UNITED STATES

## Abstract

Tactile displays provoke tactile sensations by artificially stimulating tactile receptors. While many types of tactile displays have been developed, electrotactile displays that exploit electric stimulation can be designed to be thin, light, flexible and thus, wearable. However, the high voltages required to stimulate tactile receptors and limited varieties of possible sensations pose problems. In our previous work, we developed an electrotactile display using a micro-needle electrode array that can drastically reduce the required voltage by penetrating through the high-impedance stratum corneum painlessly, but displaying various tactile sensations was still a challenge. In this work, we demonstrate presentation of tactile sensation of different roughness to the subjects, which is enabled by the arrangement of the electrodes; the needle electrodes are on the fingertip and the ground electrode is on the fingernail. With this arrangement, the display can stimulate the tactile receptors that are located not only in the shallow regions of the finger but also those in the deep regions. It was experimentally revealed that the required voltage was further reduced compared to previous devices and that the roughness presented by the display was controlled by the pulse frequency and the switching time, or the stimulation flow rate. The proposed electrotactile display is readily applicable as a new wearable haptic device for advanced information communication technology.

## Introduction

Human skin has four types of tactile receptors. These receptors, which have different properties, are located in different positions in the skin, and are responsible for different types of stimulation [1–5]. A tactile display is an interface that can provide tactile sensation to the user by stimulating these tactile receptors either electrically or by mechanically deforming the skin. Mechanotactile displays are required to be able to deform the skin either by several tens of micrometers at low frequency, or by several micrometers or even less at high frequency, depending on the receptors to be stimulated. Low-frequency and large-displacement skin deformation can be achieved by pin-type actuators using solenoids [6], while ultrasound actuators [7–9] and electrostatic actuators [10, 11] have been used to deform the skin by small displacements at high frequencies. Our group developed an array of micro-actuators consisting of hydraulic amplification mechanisms and piezo-electric actuators that can stimulate all the tactile receptors to create various tactile sensations, such as roughness and hardness [12]. To evaluate the tactile sensations that can be produced by the developed mechanotactile display, we newly proposed a sample comparison method [13]. However, the high power consumption and bulkiness of the mechanotactile displays make them unsuitable for wearable applications.

**Fig 1:**
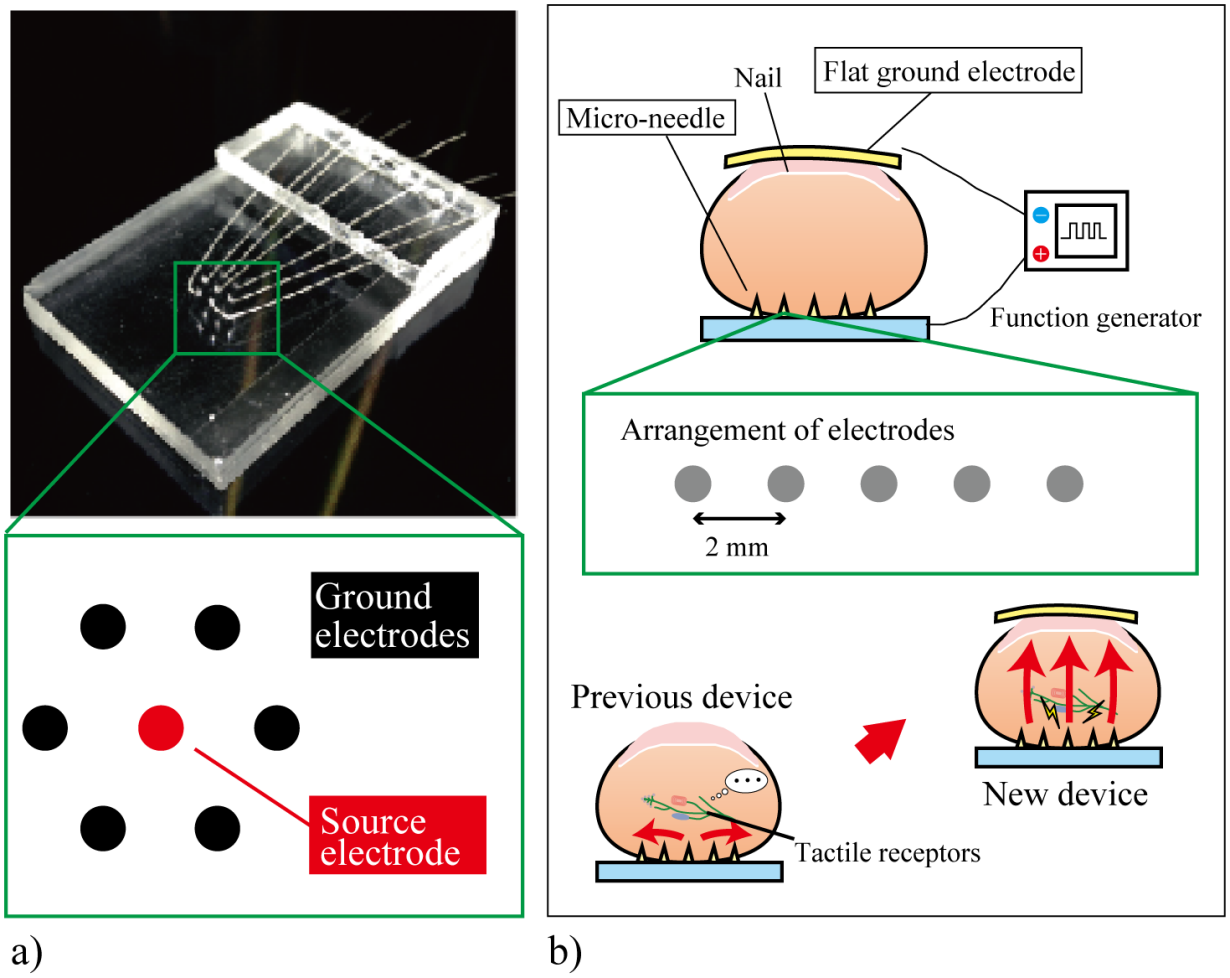
Conceptual images of the electrotactile displays. (a) Electrotactile display consisting of micro-needle electrodes [20]. The source electrode is located in the center of the electrode array, and the stimulating current only passes through the shallow region of the finger skin. (b) Newly proposed “Two-Side Needle-Flat device” with the ground electrode on the fingernail. The stimulating current passes through the finger and stimulates all the tactile receptors distributed in the skin.

Electrotactile displays stimulate the tactile receptors by applying a voltage to the skin. Arrayed flat electrodes are activated to present tactile sensations to the user [14–18]. The electrodes can be patterned onto a thin and flexible substrate, which is a huge advantage over the bulky mechanotactile displays, particularly for wearable applications. However, electrotactile displays possess two major problems; they require a high voltage of several tens of volts to stimulate the tactile receptors and can provide only a limited variety of sensations to the user that typically feel electrical and sting. The high voltages are required because of the high impedance (100 kΩ·m) of the stratum corneum, the outermost layer of the epidermis [19]. In our previous work, we developed an electrotactile display that consists of a titanium micro-needle electrode array [20]. The electrodes were manufactured to have sharp tips to penetrate through the stratum corneum, but short enough to not reach pain points. The display drastically reduced the required voltage, i.e., the threshold voltage, to create tactile sensations compared to flat-electrode-type devices. As shown in Fig 1(a), the micro-needle electrode array had seven electrodes; the electrode in the center functioned as the active electrode and the six surrounding electrodes as the ground electrodes. While the threshold voltage was successfully reduced, the tactile sensations induced by the display were still limited to electrical and stinging sensations. We considered that with this arrangement of electrodes, the stimulating currents pass through and stimulate the tactile receptors only in the shallow region of the skin. It is well-known that four types of tactile receptors are responsible for different types of stimuli and are positioned inside the skin such that they can have the highest sensitivities. Therefore, in order to create various tactile sensations, the arrangement of the electrodes needs to be changed such that all types of tactile receptors can be stimulated.

In this paper, we propose a tactile display composed of a micro-needle electrode array on the fingertip and a flat electrode on the fingernail, as shown in Fig 1(b). We call this new device the “Two-Side Needle-Flat device”. The needle electrodes are oriented to the medial-to-lateral orientation of the finger. The flat electrode functions as the ground electrode, and the stimulating currents can flow from the micro-needle electrodes to the flat electrode, i.e., they can pass through the fingers to stimulate not only tactile receptors located in the shallow regions but also those in the deep regions of the skin. Therefore, the proposed tactile display can present various tactile sensations to the subjects. We first describe the device fabrication process. Then, the threshold voltages are compared with those from previous work. Finally, the tactile sensation that can be presented by the developed display is experimentally investigated, with particular emphasis on roughness. The control parameters of the display are the pulse frequencies and stimulation time lags between the adjacent electrodes.

## Design and Fabrication

### 2.1. Design

The electrotactile display consists of micro-needles that penetrate through the high-impedance stratum corneum and stimulate the tactile receptors in the skin electrically. The lengths of the micro-needles were designed to penetrate the part of stratum corneum but not reach the pain point. The thickness of the stratum corneum varies by person. Montagna wrote that the thickness of the fingertip epidermis, including the stratum corneum, is approximately 600 μm [21]. Hirayama surveyed 50 Japanese subjects and found that the mean thickness is 663 μm (minimum: 600 μm, maximum: 765 μm) [22]. Considering that the nerves innervate the area 700 μm below the surface of the finger [21], and that needles do not penetrate completely in its length [23], we decided that the appropriate length of the micro-needles to penetrate the stratum corneum and reduce the impedance is 600 μm.

### 2.2. Fabrication

**Fig 2:**
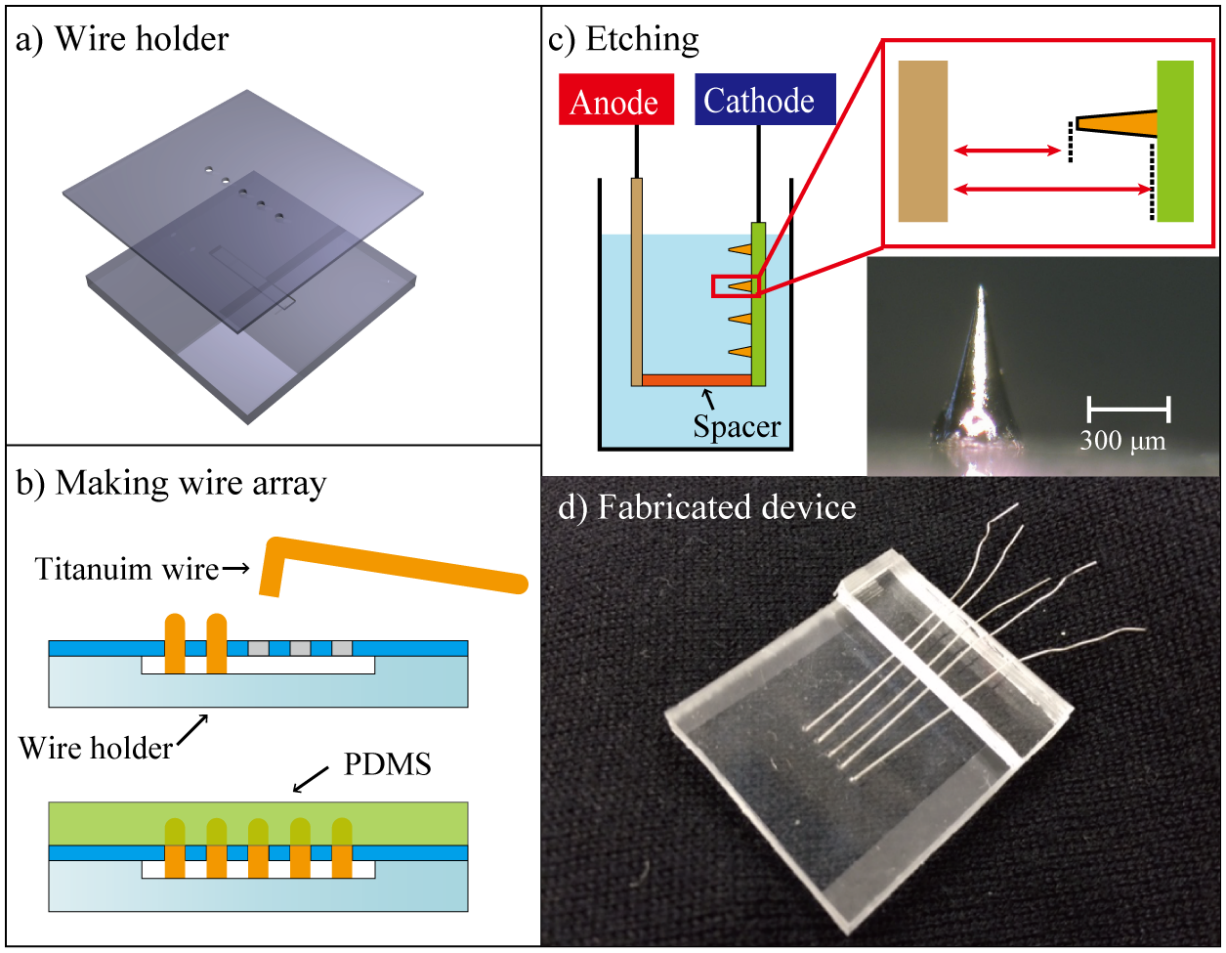
Process for fabricating the wire array. a) PMMA wire holder was mechanically fabricated. b) Titanium wires were bent and positioned into the holes of the holder, then fixed with PDMS. c) The needle shape was formed by electrochemical etching. d) Photo of the fabricated device.

First, an array of titanium wires is formed. We used 0.3-mm-diameter titanium wires (Nilaco Corp.) for the needles and polydimethylsiloxane (PDMS; SILPOT184, Dow Corning Toray) for the base of the device. Both materials are biocompatible. Fig 2(a) and 2(b) show the process for fabricating the wire array. We mechanically processed the polymethyl methacrylate (PMMA) and made the wire holder with two parts. The upper part had a thickness of 0.5 mm and five 0.31-mm-diameter holes in a row, and the lower part had an indentation of 300 μm. The initial length of the titanium wire was controlled by the depth of the indentation. The two PMMA parts were stacked and placed in a Petri dish. The titanium wires were subsequently bent and positioned into the holes of the holder. The Petri dish was then filled with PDMS casting solution and baked on a hot plate at 65°C for 4 h. The formed wire array was finally taken out of the Petri dish and released from the holder.

To sharpen the flat tips of the wires, we used electrochemical etching [24–26]. Fig 2(c) shows a schematic image of this process. The electrolyte for the wire etching consists of 4 g of sodium chloride and 60 mL of ethylene glycol solution. In our previous work [27, 28], we found that the appropriate distance between the wires (anode side) and the cathode terminal was 20 mm. We prepared a PMMA spacer 20 mm in thickness and fixed the wire array and the cathode terminal at both ends. The wire array and the spacer were set in a beaker filled with electrolyte solution. With an applied voltage of 30 V for 22 s, we obtained needles 600 μm in length and 20 μm in radius at the tip.

## Experimental Methods

### 3.1. Experimental setup

**Fig 3:**
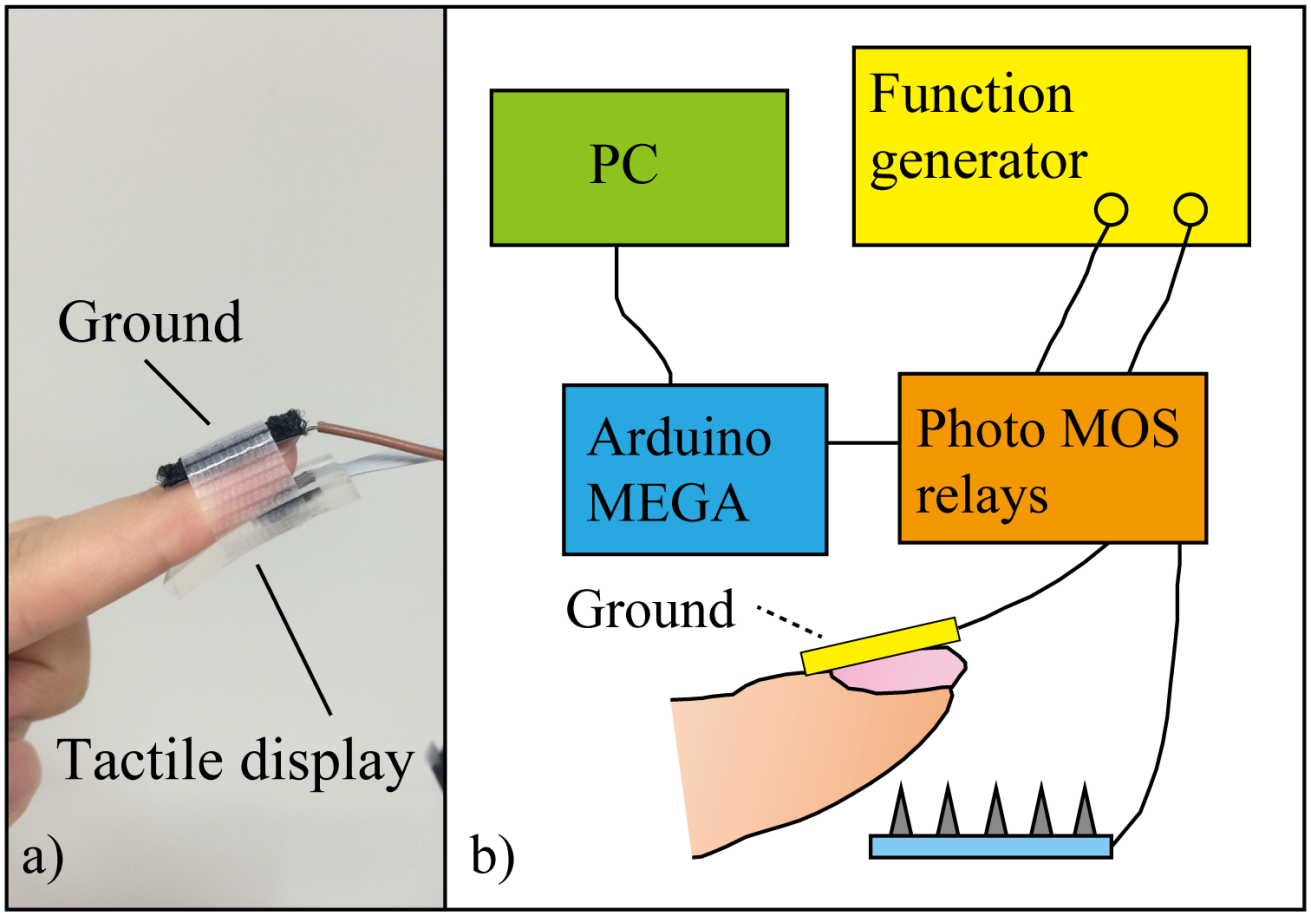
Photo of tactile display and schematic image of the experimental system.

The experimental protocol was approved by The Bioethics Board of the Faculty of Science and Technology, Keio University. The subjects received a thorough explanation of the experimental methods and then signed an informed consent form before participating in the study. The device was changed for each subject, the needles were disinfected by autoclave, and the surface of each subject's finger was disinfected with ethanol before the experiments. First, the subject was asked to mark the center of the index finger. After the needle was aligned with the marked point, the subject gently pushed the finger onto the device until the needle penetrated the finger skin. Then, the ground electrode was fixed to the fingernail with the band. The needle on the array and counter flat electrode were connected to a function generator. A photo of the tactile display attached to the finger and a schematic image of the experimental system are shown in Fig 3(a) and 3(b). Twenty subjects (16 men and 4 women; 21–25 years old) participated in the threshold voltage and voltage range experiments (sections 3.2 and 3.3). Subjects 1–5, 7, 8, 10, 11, 14 and 16 (8 men and 3 women) also participated in the perception test experiment (section 3.4).

### 3.2. Threshold voltage

We experimentally obtained the required voltages to present tactile sensations to subjects using the Two-Side Needle-Flat device. After the needle electrodes and flat electrode were attached to the subject's finger, the electric stimulus consisting of positive square-waves (duty cycle: 50%) at frequencies of 10, 20, 50 and 100 Hz were applied to the needle electrodes. For the flat-electrode device and One-Side Needle device, only the center needle was active and the others functioned as ground electrodes, but for the Two-Side Needle-Flat device, the center needle was active and the flat electrode on the fingernail functioned as the ground electrode. The applied voltage was increased from 0 V until the subject perceived a tactile sensation, which was recorded as the threshold voltage *V*_*th*_. We conducted the same experiments with the One-Side Needle device and flat-electrode device, as a reference. These devices were fabricated using the same method. Threshold voltages for the three different devices were measured three times for each frequency.

### 3.3. Voltage range

The threshold voltage *V*_*th*_ is the minimum voltage at which the subject can perceive the tactile stimulation. As the applied voltage increases, the tactile stimulation strengthens and ultimately becomes painful. We term this voltage *V*_*pain*_. The voltage that can be applied to the subject to display various tactile sensations is limited to be between *V*_*th*_ and *V*_*pain*_. A wide range, or a large difference between *V*_*th*_ and *V*_*pain*_, is preferable; however, the range is typically narrow, and due to the non-uniformity of the impedance of the finger skin, *V*_*pain*_ can vary [28]. This limits the tactile sensations that can be presented by electrotactile displays. The voltage range, *V*_*th*_ to *V*_*pain*_, was experimentally investigated for the Two-Side Needle-Flat and One-Side Needle devices. The active and ground electrodes were the same as for the threshold voltage experiment. Using the Two-Side Needle-Flat device, we also measured the voltage at which the subjects can most easily identify the tactile sensation without pain. *V*_*th*_ and *V*_*pain*_ for the two devices were measured three times for each frequency.

### 3.4. Perception test

**Fig 4:**
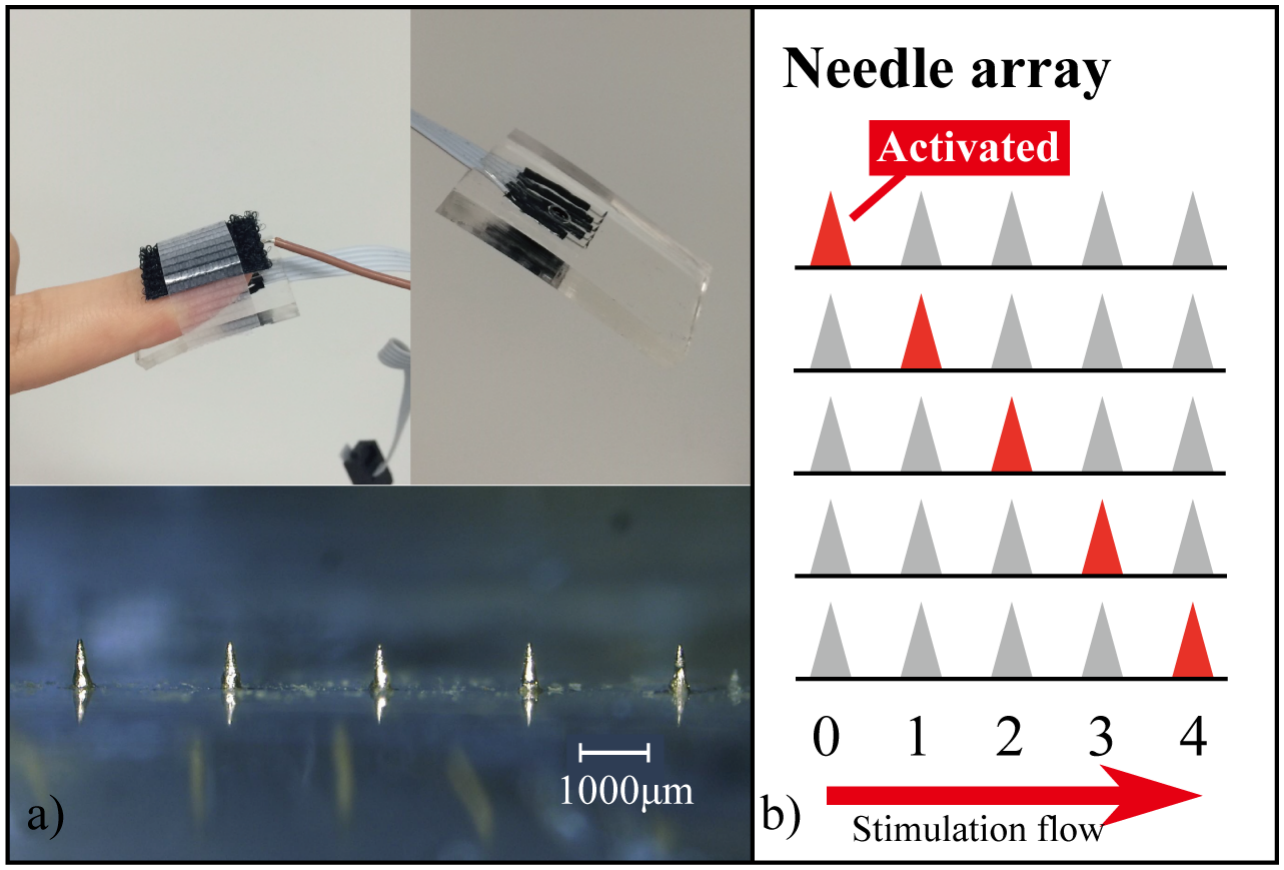
The Two-Side Needle-Flat device and schematic image of the perception test. (a) Photos of the tactile display and the needle array. (b) Illustration of the stimulation flow.

We attempted to create various tactile sensations by controlling the frequency and the peak voltage of the applied voltages, and the speed of the stimulation flow, i.e., the time lag between activation of the electrodes in series, as shown in Fig 4. Positive square pulses were generated by a pulse generator, while an Arduino MEGA micro-controller controlled the photo MOS relays to switch the active electrode at designated switching times. The signal flow was repeated in the medial-to-lateral direction of the finger. We conducted the perception test by changing the pulse frequency (10, 20, 50 and 100 Hz) and the switching time (from 30 ms to 100 ms). Each subject was asked to rate the perceived roughness on a scale from 1 (smooth) to 6 (rough). The perception test was conducted for each pulse frequency. First the stimulations with the slowest and the fastest switching times were presented as the stimuli representing the maximum and minimum of the scale, respectively. Then the sensation of each switching time was presented randomly to the subjects. The electric stimulation was applied to the subjects' fingertips until they could judge the roughness. The applied voltage was decided by the subjects so that the stimulation was clear enough to be scored but not painful. This experiment was conducted once for each subject.

## Results and Discussion

### 4.1. Threshold voltage

**Fig 5:**
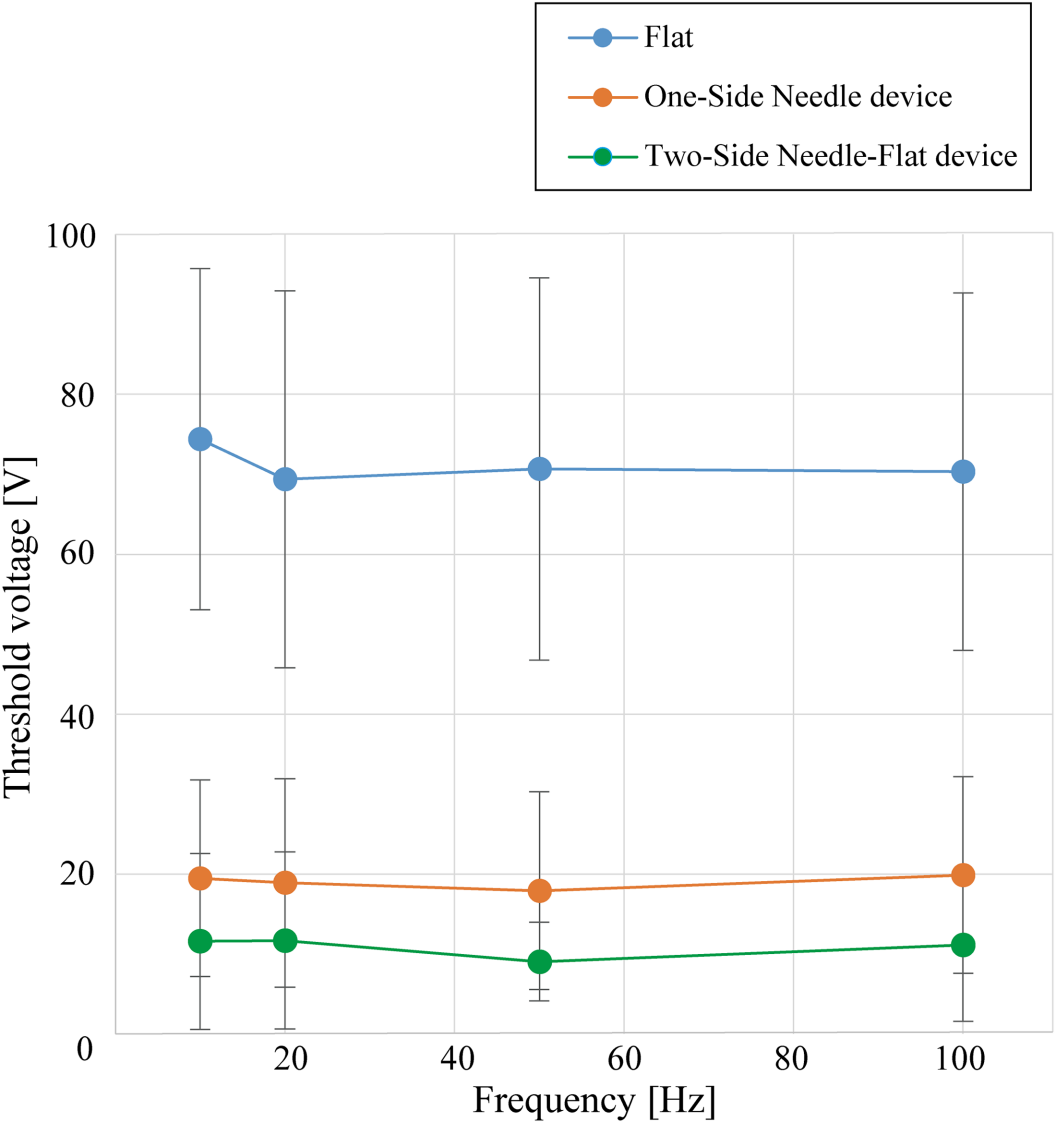
The threshold voltage of the flat-electrode device, the One-Side Needle device, and the newly proposed Two-Side Needle-Flat device with respect to the pulse frequency. The results shown are for 17 subjects. The error bars represent standard deviations.

**Fig 6:**
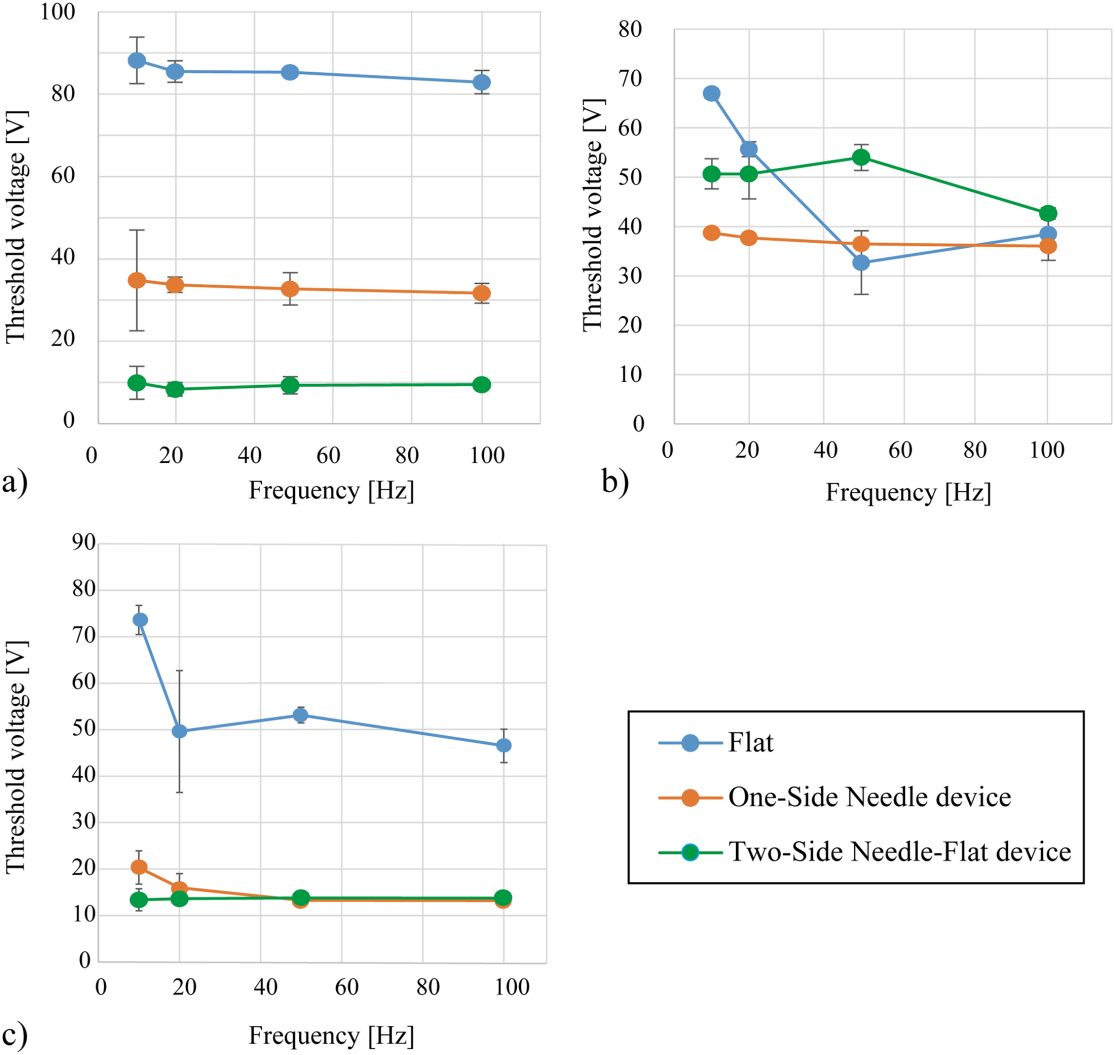
Results for three subjects. a) Statistically significant results for one subject, p < .05. b) The threshold voltage of the Two-Side Needle-Flat device is bigger than that of the One-Side Needle device, and the difference in threshold between the flat-electrode device and the Two-Side Needle-Flat device is not statistically significant. c) No significant difference in threshold between the Two-Side Needle-Flat device and the One-Side Needle device.

Fig 5 shows the threshold voltage of the flat-electrode device, the One-Side Needle device, and the proposed Two-Side Needle-Flat device with respect to the pulse frequency. The figure shows the averages for 17 subjects. Three of the 20 subjects could not feel any sensation with the needle devices, so their results were excluded. As a trend, the threshold voltage of the newly proposed Two-Side Needle-Flat device was found to be even smaller than that of the previous One-Side Needle device and much smaller than that of the flat electrodes. The dependent t-test was used to assess the statistical significance of this trend, and showed that the differences in the mean threshold voltage between the flat-electrode device (*M* = 72.29, *SD* = 21.57) and Two-Side Needle-Flat device (*M* = 11.15, *SD* = 10.81) across the whole subject were statistically significant (t(16) = 9.82, p < .05, d = 3.58). In the same way, the mean threshold voltage of the One-Side Needle device (*M* = 19.03, *SD* = 11.76) and that of the Two-Side Needle-Flat device were compared. The result of the dependent t-test showed that the differences in the mean threshold voltage of these two devices across the whole subject were statistically significant (t(16) = 2.97, p < .05, d = .70). The mean difference in threshold between the One-Side Needle device and the Two-Side Needle-Flat device across the subjects was 7.88 V > 0 (*SD* = 10.95), this also shows that the Two-Side Needle-Flat device requires less voltage than the One-Side Needle device. With the above statistical analyses we verified that the threshold voltage of the newly proposed Two-Side Needle-Flat device was significantly smaller than that of the One-Side Needle device. Then the results of each subject were analyzed independently with the dependent t-test. This result revealed that 12 subjects showed a threshold voltage of the Two-Side Needle-Flat device that was significantly smaller than their threshold voltage of the One-Side Needle device (p < .05), 4 subjects showed a threshold voltage of the Two-Side Needle-Flat device that was significantly bigger than their threshold voltage of the One-Side Needle device (p < .05), and 1 subject showed no significant difference in threshold voltage between the two devices. Fig 6(a) shows the results for a subject where the threshold voltage of the Two-Side Needle-Flat device is significantly smaller than that of the other two devices (p<0.05). Fig 6(b) shows the results for a subject in which the threshold voltage of the Two-Side Needle-Flat device is bigger than that of the One-Side Needle device and there is no significant difference between the flat-electrode device and the Two-Side Needle-Flat device. Fig 6(c) shows results for a subject where there is no significant trend between the Two-Side Needle-Flat device and the One-Side Needle device. The threshold voltage varied among subjects because of the thickness of the stratum corneum, the condition of the skin, and, in particular, the state of the inserted needles. Good insertion of the needles is the most important condition to sufficiently decrease the threshold voltage of the Two-Side Needle-Flat device.

This experiment revealed that the newly proposed Two-Side Needle-Flat device requires less voltage to produce a tactile sensation. The smaller voltage, which equates to low power consumption, is of great benefit for wearable applications. We consider that the Two-Side Needle-Flat device can stimulate the tactile receptors deep in the finger skin by having the counter ground electrode on the fingernail. This was implied by the fact that the subjects experienced tactile sensations with less stinging. The threshold voltage did not vary with respect to the frequency.

### 4.2. Voltage range

**Fig 7:**
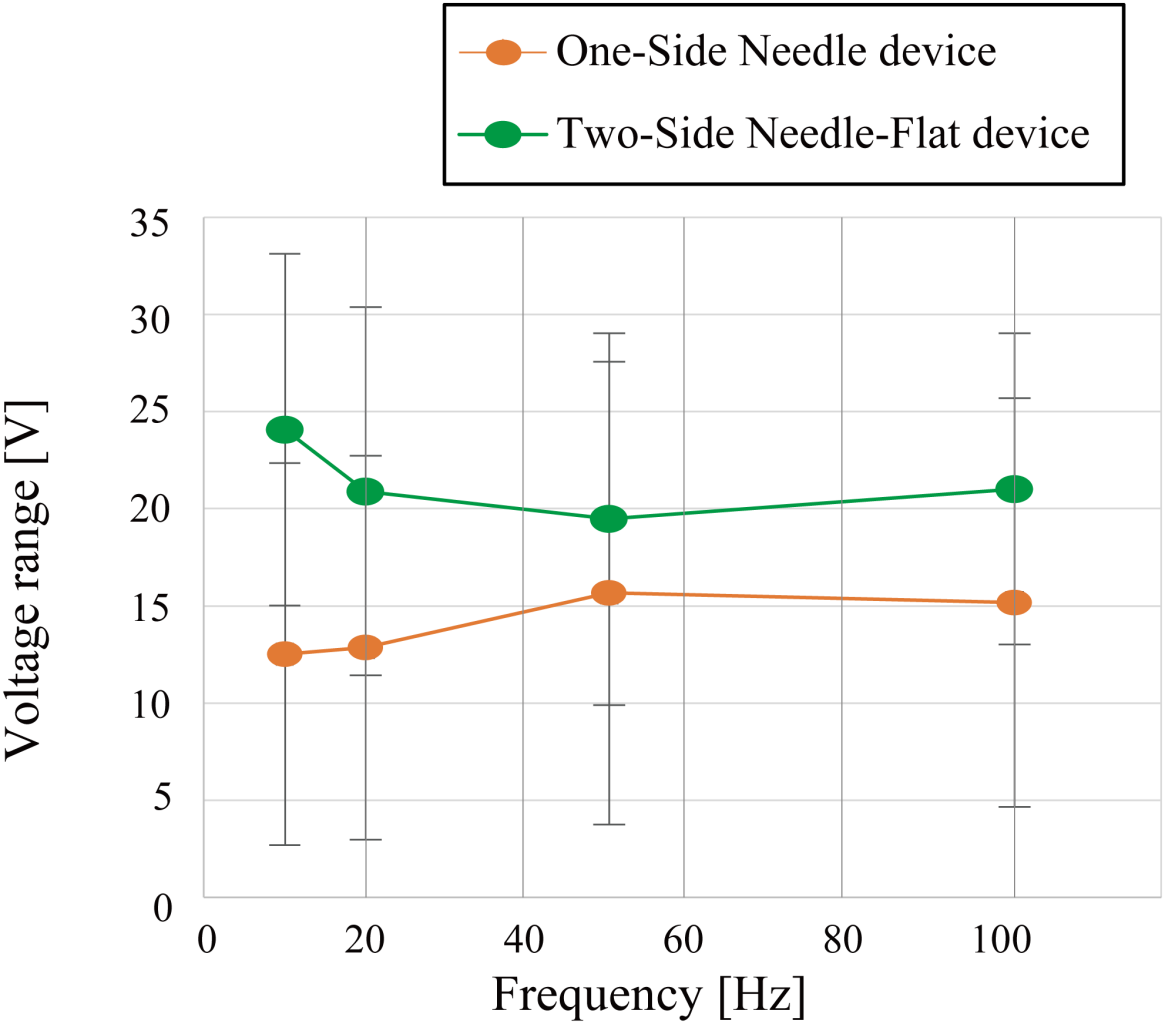
The difference between the threshold voltage and the painful voltage for the One-Side Needle and Two-Side Needle-Flat devices with respect to the frequency. The error bars represent standard deviations. The results shown are for 17 subjects. The range did not change with the frequency, but the Two-Side Needle-Flat device had a wider range.

Fig 7 shows the difference between *V*_*th*_ and *V*_*pain*_; data are the averages for 17 subjects. The voltage range of the Two-Side Needle-Flat device was greater than that of the One-Side Needle device. The dependent t-test was used to assess this trend and showed that the difference in voltage range between the Two-Side Needle-Flat device (*M* = 21.37, *SD* = 7.77) and the One-Side Needle device (*M* = 14.05, *SD* = 9.67) was statistically significant across the whole subject (t(16) = 2.99, p < .01, d = .83). The mean difference between the voltage range of the Two-Side Needle-Flat device and that of the One-Side needle device was 7.31 V and the standard deviation was 9.78 across the subjects. These results reveal that the voltage range of the Two-Side Needle-Flat device is significantly wider than that of the One-Side Needle device. Then the statistical analysis was conducted for the results of each subject. The result of the dependent t-test revealed that 10 subjects showed a voltage range of the Two-Side Needle-Flat device that was significantly wider than that of the One-Side Needle device (p < .05), 6 subjects showed no significant difference in voltage range between the two devices, and 1 subject showed a voltage range of the Two-Side Needle-Flat device that was narrower than that of the One-Side Needle device (p < .05). The narrow range of the One-Side Needle device resulted in limited varieties of tactile sensations. In addition, the subjects were more likely to feel pain with the One-Side Needle device. The newly proposed device with a ground electrode on the fingernail was verified to have a wider voltage range, which broadens the range of tactile display experiments that can be conducted.

**Fig 8:**
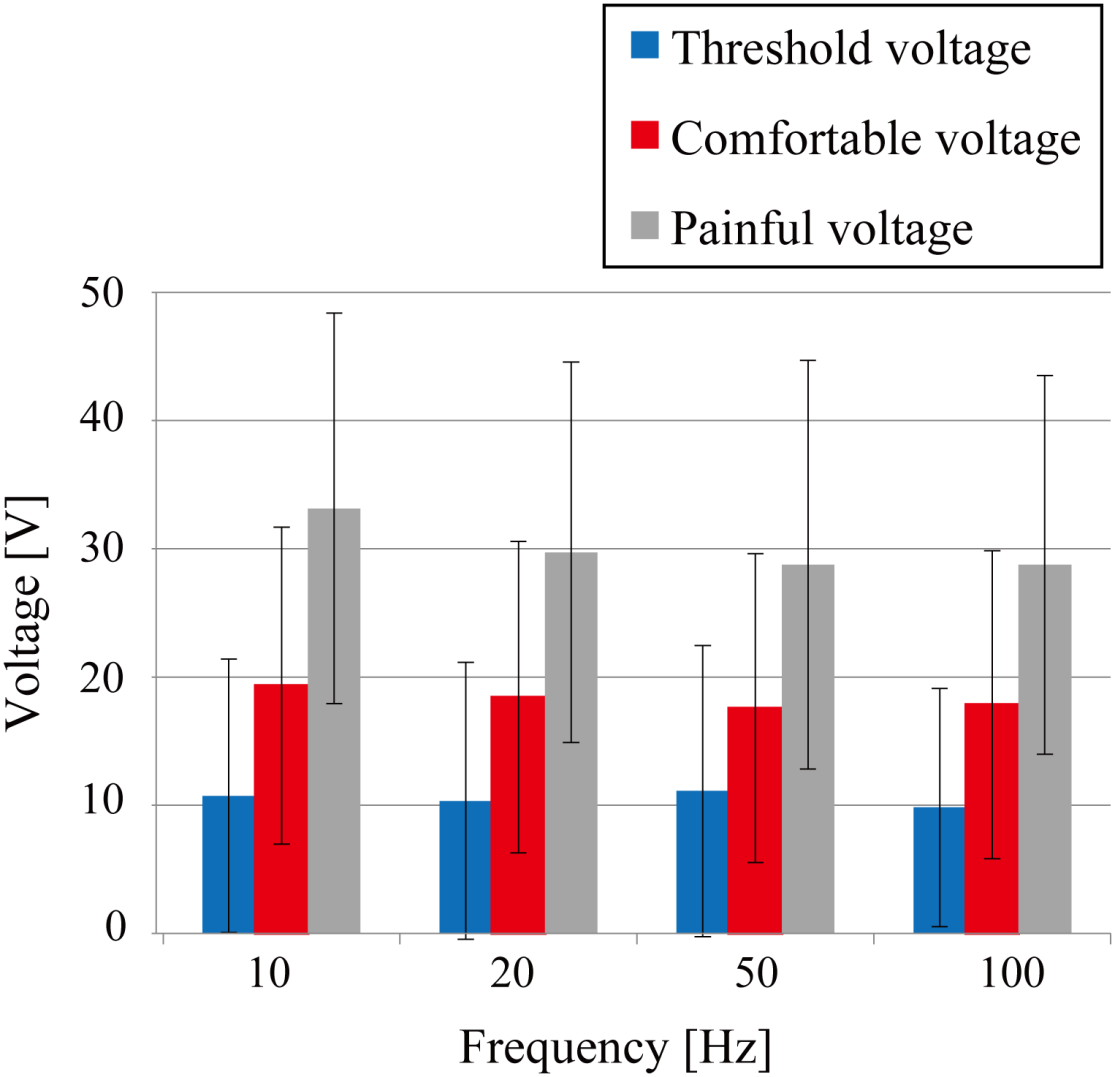
Relationship between the comfortable voltage, threshold voltage and painful voltage of the Two-Side Needle-Flat device. Data are averages for 17 subjects.

Fig 8 shows the relationship between the threshold voltage *V*_*th*_ the painful voltage *V*_*pain*_ and the comfortable voltage of the Two-Side Needle-Flat device; data are the averages for 17 subjects. The value of the comfortable voltage was approximately halfway between *V*_*th*_ and *V*_*pain*_. The large margin to reach the painful voltage allows for stable presentation of tactile sensations without the risk of pain due to any sudden change in skin conditions.

### 4.3. Perception test

**Fig 9:**
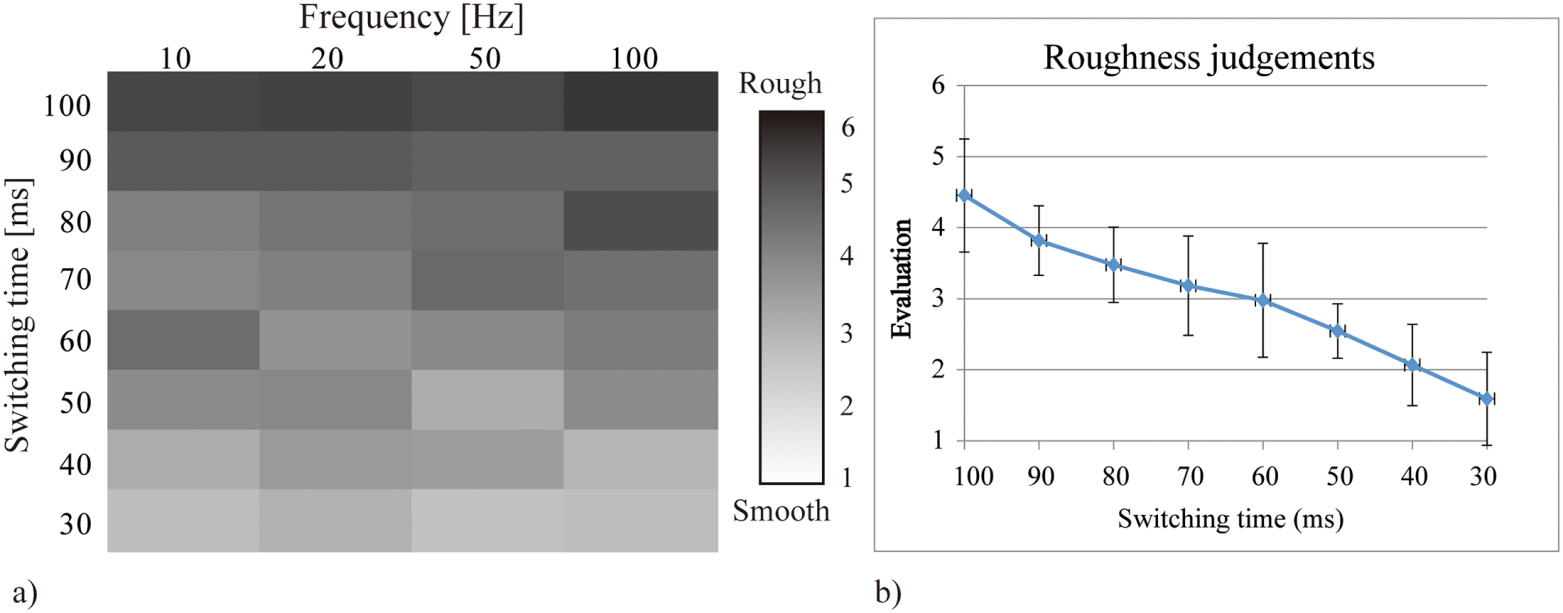
Results of the perception test. a) The grayscale blocks correspond to the averages of the roughness reported by 11 subjects. b) The averages of the roughness judgements (averaged across frequencies) for 11 subjects. The error bars represent the standard deviations.

Fig 9 shows the results of the perception tests. The grayscale blocks in Fig 9(a) correspond to the averages of the roughness judgements for 11 subjects (1 = white and 6 = black), with respect to the switching time and frequency. The trend clearly indicates that the roughness increases with the switching time, as the blocks darken toward the top of the chart. Correlation analysis was performed to verify this trend. The average roughness judgments across subjects and the switching time have a positive correlation (r = .993, p < .01). The frequency is not a dominant factor. This result verified that we can represent different sensations by changing the switching time.

**Fig 10:**
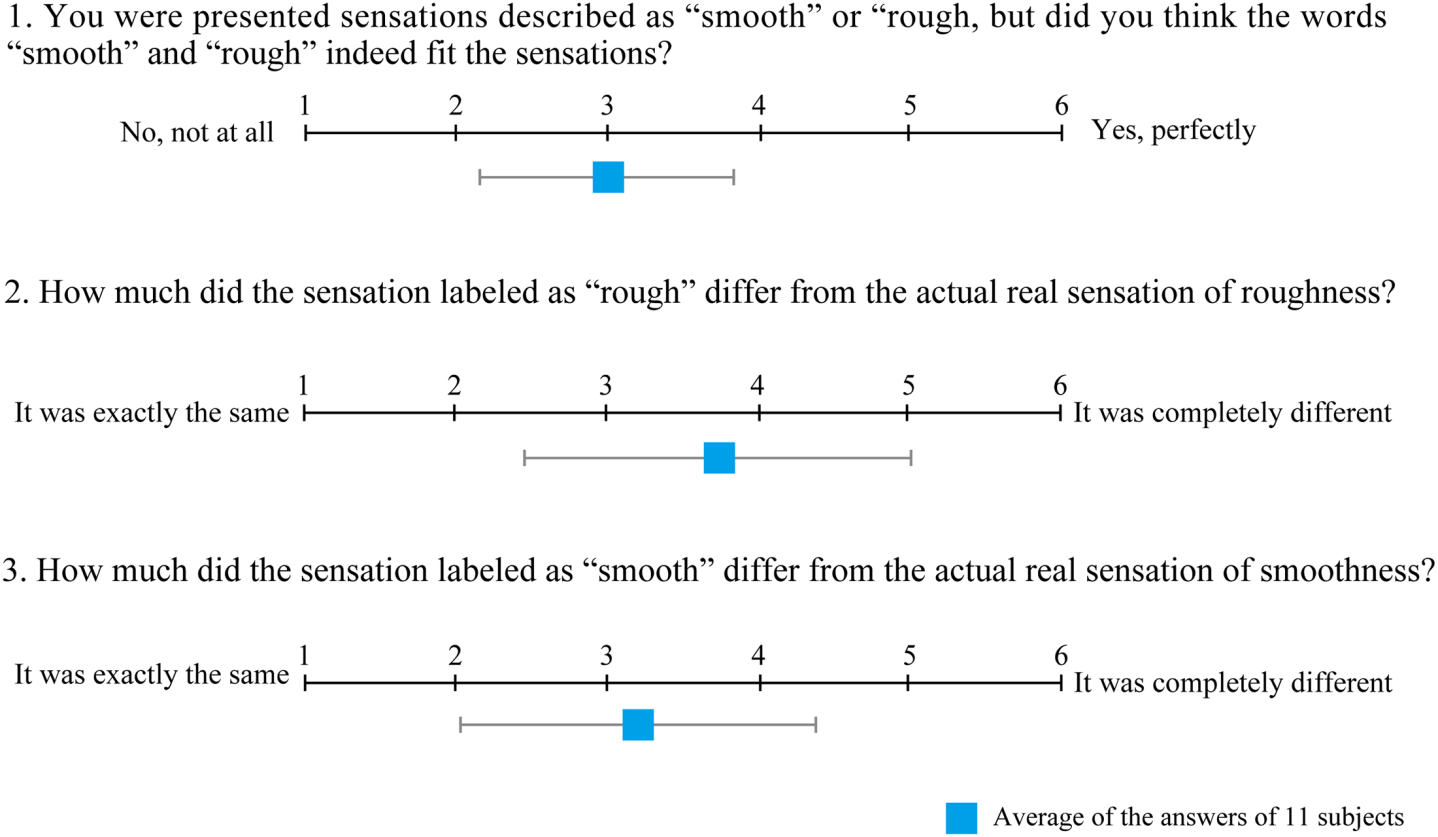
Questionnaire results. The questionnaire items and the average of the answers of 11 subjects with the standard deviations.

After the perception tests, we asked the subjects to complete a questionnaire about the sensation that they perceived. Fig 10 shows the items on the questionnaire and the answers of the 11 subjects. We also encouraged the subjects to provide feedback regarding each question. The remarks of the subjects revealed they recognized that the sensations changed with the switching time, but the presented sensations were not the same as a real rough or smooth sensation. The sensation was a “slow-quick tingling” or “tickle” sensation rather than a real “rough-smooth” sensation for some subjects. Most of the subjects reported that the absence of the motion of the finger prevented them from feeling like they were touching something, and that the “smooth” sensation could be closer to the real one than the “rough” sensation, but the sensation presented by five needles was too short to recognize it perfectly. This is one of the reasons why the score of the question 3 is situated in the middle of the score. To make it feel as if subjects were touching a real surface, another system that has several lines of needles covering a larger area of the finger would be required. We also found that a uniform insertion of five needles was indispensable to perfectly perceive the difference in sensations for each switching time. When one needle provided a stronger stimulation than the other needles, the subjects had difficulty judging the roughness correctly. Further study and improvement of the system are necessary to represent real roughness perception; however, we have successfully presented different sensations using our new device.

## Conclusions

An electrotactile display with micro-needle electrodes on the fingertip and a counter flat electrode on the fingernail was proposed and demonstrated. The device is capable of stimulating the tactile receptors located in deep regions as well as in shallow regions of the skin, which enabled an even lower threshold voltage for displaying tactile sensations to subjects. Moreover, it was experimentally verified that the difference between the threshold voltage and painful voltage was larger for the new device compared to a previous device, which allows more variation of the stimulating signals and thus, a tactile sensation. The new device could successfully control the roughness of the presented tactile sensation by activating individual electrodes in series. The proposed electrotactile display is advantageous in terms of its low power consumption, light weight and flexibility, and is readily applicable to innovative information communication technologies using tactile stimulation.
